# Boosting of Waned Humoral and Cellular Responses to SARS-CoV-2 Variants of Concern Among Patients with Cancer

**DOI:** 10.1158/2767-9764.CRC-22-0298

**Published:** 2022-11-17

**Authors:** Duncan R. McKenzie, Rosalind Graham, Thomas Lechmere, Clara Domingo-Vila, Thanussuyah Alaguthurai, Celeste Arman, Emily Pollock, Charalampos Gousis, Helen Kakkassery, Esme Carpenter, Ashwini Kurshan, Jennifer Vidler, Austin Kulasekararaj, Piers Patten, Bernard V. North, Timothy Tree, Katie J. Doores, Adrian C. Hayday, Sheeba Irshad

**Affiliations:** 1 The Francis Crick Institute, London, United Kingdom; 2 Comprehensive Cancer Centre, School of Cancer & Pharmaceutical Sciences, King’s College London, London, United Kingdom; 3 Department of Infectious Diseases, School of Immunology & Microbial Sciences, King’s College London, London, United Kingdom; 4 Peter Gorer Department of Immunobiology, School of Immunology and Microbial Sciences, King’s College London, London, United Kingdom; 5 Breast Cancer Now Research Unit, King’s College London, London, United Kingdom; 6 Guy’s and St Thomas’ NHS Foundation Trust, London, United Kingdom; 7 Department of Haematological Medicine, King’s College Hospital, London, United Kingdom; 8 Clinical Trials Unit, King’s College London, London, United Kingdom; 9 Cancer Research UK (CRUK) Clinician Scientist, London, United Kingdom

## Abstract

This study offers longitudinal insight into the impact of three SARS-CoV-2 vaccinations on humoral and cellular immunity in patients with solid cancers, patients with hematologic malignancies, and persons without cancer. For all cohorts, virus-neutralizing immunity was significantly depleted over a period of up to 9 months following the second vaccine dose, the one striking exception being IL2 production by SARS-CoV-2 antigen-specific T cells. Immunity was restored by the third vaccine dose, except in a substantial number of patients with hematologic malignancy, for whom both cancer type and treatment schedule were associated with nonresponse. Thus, whereas most patients with myelodysplastic syndrome were conspicuously good responders, some patients with other hematologic malignancies receiving cancer therapies within 2 weeks of vaccination showed no seroconversion despite three vaccine doses. Moreover, SARS-CoV-2 exposure during the course of the study neither prevented immunity waning, even in healthy controls, nor guaranteed vaccine responsiveness. These data offer real-world human immunologic insights that can inform health policy for patients with cancer.

## Introduction

We previously reported that very many patients with cancer are incompletely protected to SARS-CoV-2 infection after an initial dose of Pfizer-BioNTech BNT162b2 COVID-19 vaccine (1), and that many patients with hematologic cancer failed to seroconvert even after a two-dose vaccine schedule (2). Since then, immunocompromised patients, including those with an active cancer diagnosis, have been globally prioritized for accelerated receipt of COVID-19 vaccine boosters. Specifically, seroconversion rates after two COVID-19 vaccine doses were reported as 99% [95% confidence interval (CI), 98–100] for people who were not immunocompromised, 92% (CI, 88–94) for patients with solid cancer (SC), and only 64% (CI, 50–76) for patients with hematologic cancers (3). In September 2021, the Joint Committee on Vaccination and Immunisation in the United Kingdom issued guidance to offer a third dose of either the Moderna (SpikeVax) or Pfizer-BioNTech BNT162b2 COVID-19 vaccine for the immunocompromised (4). While reports attest to the fact that many patients with a weaker immune response can benefit from a third vaccine dose, it was also reported that 44% of patients with hematologic cancer continued to fail to mount clear serologic responses (5). Data from Israel also showed waning efficacy in the general population after about 4 months following two COVID-19 vaccine doses (6), and long-term follow-up of vaccine trial participants also revealed a growing risk of breakthrough infection (7).

As outlined in our recent review of data (8), studies reporting responses to vaccines in patients with cancer show confounding heterogeneity in terms of patient populations and with respect to the experimental assays used. This heterogeneity complicates meta-analyses of similar patient groups. Thus, there remains uncertainty over: (i) the durability of vaccine-induced SARS-CoV-2–specific immunity; (ii) the immunogenicity of booster vaccinations in patients with cancer; and (iii) the impact of emerging variants of concern (VoC). Thus, we have undertaken a further investigation of participants in the SOAP (Sars-CoV-2 fOr cAncer Patients) study, a prospective, longitudinal cohort of patients with cancer, with first study recruitment on December 8, 2020. Specifically, we have: (i) clarified in patients with cancer the degree of waning of antibodies, of serum neutralization titres against the parental strain and VOCs, and of T-cell responses; (ii) evaluated the immune responses of patients with cancer to a third dose of COVID-19 vaccine; and (iii) provided safety data following the third dose of the BNT162b2 COVID-19 vaccine in patients with cancer. In addition, for a subset of the cohort, data captured from earlier timepoints in the SOAP study facilitated analysis of the kinetics of vaccine responses over time, a key and unique aspect of this study by comparison with other reports.

## Materials and Methods

### Study Design and Participants

Patients with a known diagnosis of cancer presenting at three London hospitals, who were eligible for COVID-19 vaccines were screened and approached for written informed consent into the SOAP study, a prospective longitudinal observational study of cancer patients. Early in the vaccination program, we included a cohort of prioritized healthy controls (HC; mostly health care workers) so that we could benchmark the effectiveness of local vaccination protocols and our experimental methods with other COVID-19 vaccination studies of healthy individuals. The trial was approved by the Institutional Review Boards of the participating institutions (IRAS ID: 282337 REC ID: 20/HRA/2031) and was conducted in accordance with the recognized ethical guidelines of Declaration of Helsinki.

### Study Procedures

Previous study patients (1, 2) and newly recruited patients were followed up for further blood sampling prior to the third dose [timepoint 5 (TP5)] and 3 weeks after the third vaccine dose of the BNT162b2 vaccine (TP6). Telephone consultations to evaluate reactogenicity and safety of the third dose were conducted approximately 10 days postinoculation. Adverse events were graded according to the scale: mild, does not interfere with activity; moderate, interferes with activity; severe, prevents daily activity; and grade 4, emergency department visit or hospitalization. Full details of the patient inclusion and exclusion criteria are in the previously published protocol (1).

### Laboratory Analyses

The serologic neutralization assays were performed as described previously (1, 9). IgG binding was measured against recombinant Wuhan spike (9). HIV-1–based virus particles pseudotyped with SARS-CoV-2 Wuhan strain wild type (WT), delta (B.1.617.2), and omicron (BA.1) spikes were used to measure neutralization of infection of HeLa cells expressing the ACE2 virus receptor. The omicron (BA.1) spike plasmid was obtained from Prof. Wendy Barclay, Imperial College London.

Similar to previous studies (1), fluorospot assays were used to quantitate T cells secreting IFNγ or IL2, or both, in response to stimulation with peptide mixes presented on autologous antigen-presenting cells: SARS-CoV-2 spike 2 peptides [PepMix SARS-CoV-2 (spike glycoprotein)]; SARS-CoV-2 RBD (receptor-binding domain) peptides (“RBD”); and control peptides derived from cytomegalovirus (CMV), epstein-barr virus (EBV), flu, and tetanus (“CEFT”).

On the basis of methodology used in ref. 10, we investigated the waning cellular immune response induced by vaccination by stimulating the peripheral blood mononuclear cells (PBMC) harvested at TP5 to peptide pools [PepMix SARS-CoV-2 (spike glycoprotein)]. We detected expression of surface activation-induced markers (AIM) on CD4 T cells, defined by upregulation of CD40 L and CD69, and CD8 T cells, defined by CD137 and CD69 upregulation (10, 11). Antigen-specific responses were quantified on the basis of the frequency of AIM^+^ T cells in stimulated samples above background frequencies in paired unstimulated controls, with antigen-specific AIM^+^ CD4^+^ T cells. We further characterized the differentiation status of antigen-specific T cells induced by vaccination using CCR7 and CD45RO differentiation markers to define naïve (N; CCR7^+^CD45RO^−^), central memory (CM; CCR7^+^CD45RO^+^), effector memory (EM; CCR7^−^CD45RO^+^) and terminally differentiated effector (TEMRA; CCR7, CD45RO double negative) populations.

### Statistical Analysis

The sample size was not based on statistical hypothesis testing. All participants with available data were included in the safety and immunogenicity analyses, with the exception of those suspected of SARS-CoV-2 infection as detailed in the Results. Samples were immediately assigned an ID upon receipt, and sample processing and analysis was done without any experimental operator knowing the nature of the sample, consistent with good laboratory practice. Statistics were computed in R, version 4.0.0 (R Core Team 2020), using rstatix (version 0.7.0) and, for partially matched Wilcoxon tests, robustrank (version 2019.9–10). Statistical tests and *P*-value corrections were performed on log-transformed data as detailed in figure legends. The significance threshold for *P* values was less than 0.05 after correction for multiple comparisons. Nonsignificant *P* values are not reported in figures. The proportions of responders above the threshold and 95% CIs calculated by the Wilson method are reported. Serologic responders were defined as >70 EC_50_ dilution for anti-spike IgG. T-cell responders were defined as ≥7IFNγ^+^ and/or IL2^+^ spots per 10^6^ PBMC in response to RBD and/or S2 peptide pools. Combined serologic and T-cell responders were those who mounted both response measures by these definitions. The trial is registered with the NHS Health Research Authority (HRA) and Health and Care Research Wales (REC ID: 20/HRA/2031).

### Data Availability Statement

Data are available from the authors upon reasonable request.

### Ethical Statement

The study was approved by the institutional review boards of the participating institutions (IRAS ID: 282337 REC ID: 20/HRA/2031). Participants who were eligible for the study were screened and approached for written informed consent into the SOAP study.

## Results

### Patient Demographics and Baseline Characteristics

**Figure 1:**
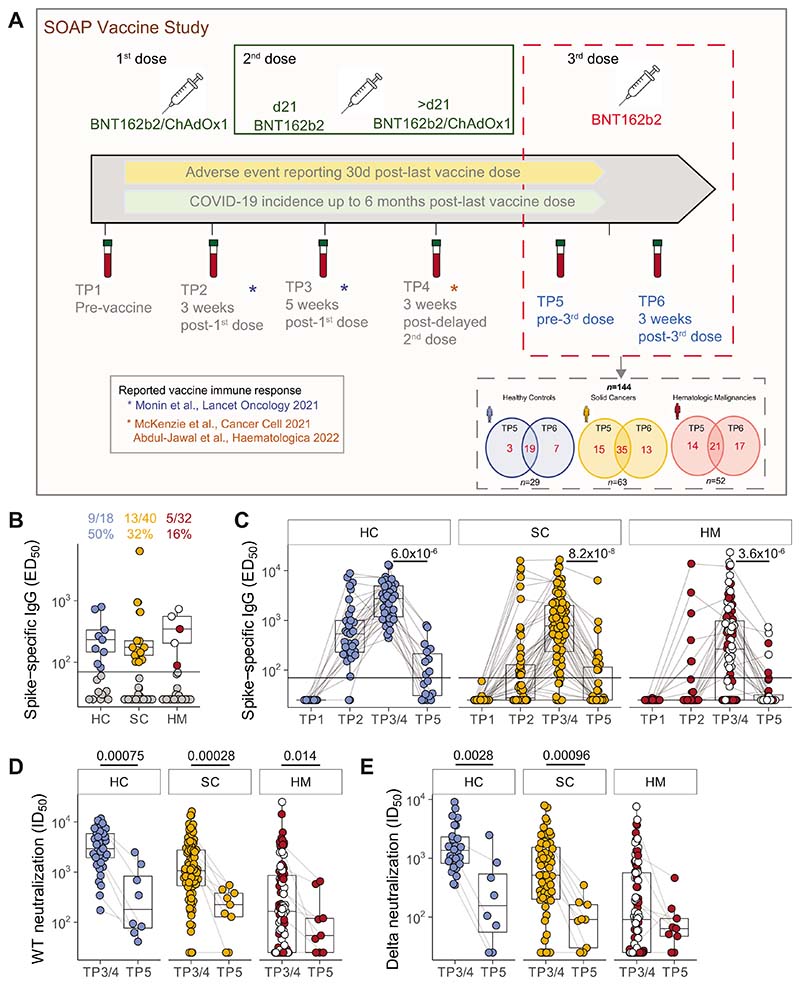
Waning serologic responses to SARS-CoV-2 vaccination. **A,** Study design. Venn diagrams demonstrated the number of patients recruited for TP5 and TP6 analysis, as well as patients with both timepoints available for analysis. **B,** Plasma spike-specific IgG titres at TP5. Numbers represent the frequency of individuals with titres above the seropositivity threshold (>70 EC_50_ dilution). Sample comparisons tested by a Kruskal–Wallis test with Dunn multiple comparisons test and corrected by the Holm method (ns). Boxplots and statistics summarize responder values only (HC *n* = 9/18, SC *n* = 13/40, HM *n* = 5/32). Gray: nonresponders; blue: HC, orange: SC, red: non-MDS HM, white: MDS HM. **C,** Spike-specific IgG titres in plasma samples at TP1–5. Patient-matched samples are linked by lines. Matched samples at TP3/4 and TP5 (HC *n* = 15, SC *n* = 37, HM *n* = 31) were compared by a Wilcoxon signed-rank test and *P* values corrected by the Benjamini–Hochberg method. White: MDS. Plasma neutralization titres against wildtype (**D**) and B.1.617.2 delta (**E**) SARS-CoV-2 variants at TP3/4 and TP5. Sample comparisons tested by a partially matched Wilcoxon test and *P* values corrected by the Benjamini–Hochberg method (WT TP3/4: HC *n* = 37, SC *n* = 83, HM *n* = 93; delta TP3/4 *n* = 26, SC *n* = 64, HM *n* = 88; WT/delta TP5 *n* = 8, SC *n* = 9, HM *n* = 9). White: MDS. Boxplots represent the median, Q1 and Q3. Horizontal lines represent response thresholds. ED_50_: plasma dilution at 50% binding; HC: healthy control; HM: hematologic malignancy; ID_50_: inhibitory dilution at which 50% of virus particles are neutralized; MDS: myelodysplastic syndrome; ns: nonsignificant; SC: solid cancer; TP: timepoint.

From December 8, 2020, until December 21, 2021, 187 patients with cancer [101 patients with SC and 86 patients with hematologic malignancy (HM)] and 44 HCs consented to enrolment in the SOAP-vaccine study. Previous study participants (1, 2) and newly recruited patients were followed up for further blood sampling prior to the third dose(TP5) and at approximately 3 weeks after the third vaccine dose (TP6; Fig. 1A). Samples and data obtained up until November 2021 were analyzed. Samples from 144 patients were available for analysis at TP5 and/or TP6; with 75 of these patients having a sample available at both timepoints (19 HC, 35 SC, 21 HM; Fig. 1A). A total of 107 samples (22 HC, 50 SC, 35 HM) were evaluable at TP5 while 112 samples (26 HC, 48 SC, 38 HM) were evaluable at TP6. The clinical characteristics of all 144 trial participants at TP5/TP6 are shown in Table 1. Most patients had received two primary doses of the BNT162b2 COVID-19 vaccine, but approximately 12% (17/144) had received the viral vector vaccine (ChAdOx1 nCov-19; AstraZeneca) as the first and/or second dose. All 17 of these patients were patients with a confirmed diagnosis of myelodysplastic syndrome (MDS).

The major comparisons for patients with cancer were based on the longitudinal evolution of their own responses to vaccination, as judged by multiple immunologic metrics and safety. The distribution of the anticancer treatments given in relation to the date of the third vaccine dose for SCs and HMs is shown in Supplementary Table S1. A total of 42% (20/48) of patients with SC received anticancer treatment within 15 days preceding the third dose, and 48% (23/48) received anticancer treatment within 15 days following the third dose, among whom 15 patients received treatment within 15 days before and after treatment. For patients with HM, 39% (15/38) received anticancer treatments within 15 days preceding the delayed third dose, and 37% (14/38) received anticancer treatment within 15 days following the delayed third dose, among whom 13 received treatment within 15 days both before and after treatment (Supplementary Table S1).

### Significant Waning of Serologic and Neutralization Responses Over Time

As described previously (1, 2, 11), prior infection can confound attempts to measure vaccine efficacy. Therefore, we excluded 17 of the 107 subjects evaluable for analysis at TP5 (4 HC, 10 SC, and 3 HM) from the overall immune efficacy analysis on the basis of PCR-confirmed infection, or detectable anti-spike IgG titre at baseline (TP1) and/or anti-N IgG titre at any TP (note that N was not included in the vaccines). Data from these patients are returned to later (see below). Their exclusion left a non–virus-exposed cohort of 90 individuals for analysis at TP5.

Median (Q1, Q3) times (days) from the second vaccine dose to blood sampling at TP5 prior to the third dose were 215.5 (200–259) days for HCs; 187 (180–213) days for patients with SC; and 188 days (180.5–196) for patients with HM (Table 1). For non–virus-exposed individuals, positive anti-S IgG titres across the three cohorts at TP5 were observed in only 50% (9/18) of HCs, 32% (13/40) of patients with SC, and 16% (5/32) of patients with HM (Fig. 1B; Table 2) [Note, patients with HM presenting with MDS are identified as white circles in all figures]. Although we had previously reported that ChAdOx1 induced weaker humoral and cellular vaccines responses than those induced by BNT162b2 responses in patients with MDS (12), further evaluation across these comparative groups was not possible due to limitations in the number of samples (footnote in Table 1). Median titres among responders were comparable across all three cohorts (Fig. 1B). These positive response rates at TP5 contrast strikingly with the serological response data following two doses of vaccine; data which were captured at either TP3 (blood sampling 2 weeks following the second dose given at day 21 following the primary inoculum) or TP4 (blood sampling at 3 weeks following a delayed (21–84 days) second dose: those response rates were: 100% for HC (38/38); 87% for SC (72/83), and 61% for HM (57/93; Fig. 1C; Table 2). Among those individuals with evaluable samples at both TP3/4 and TP5, we observed a decline in the median anti-S IgG across all three cohorts (HC, from 2735.0 to 71.0; SC, from 775.9 to 25.0;HM, from 264.0 to 25.0, *P*-values HC 6 × 10^−6^,SC8 × 10^−8^,HM4 × 10^−6^; Fig. 1C). Such striking declines are consistent with those reported for the general population (13).

Among those who were serologic responders following completion of the two-dose vaccine schedule, the number of patients and HCs who fell below the anti-S IgG detection cutoff were: 8/15 HC (53%), 19/37 SC (51%), and 15/31 HM (48%; Fig. 1C; Supplementary Table S2). In addition, 6 of 31 patients with SC and 11 of 31 HMs had scored as nonresponders at 2 weeks following two doses and remained as nonresponders up to and including TP5 (Supplementary Table S2). The waning serologic response across the three cohorts was not significantly associated with age, or any specific cancer type in the cancer patient population (Supplementary Fig. S1A–C). Given that our enriched population of patients with MDS HM (40% of all patients with blood cancer at TP5) demonstrated comparatively higher immunogenicity of the COVID-19 vaccine, we also considered these patients separately (Supplementary Fig. S1D), and yet the waning serologic response was comparable across both cohorts (Supplementary Fig. S1C). In addition, we did not observe any significant association of waning serologic responses with having had antitumor and/or cytotoxic or steroid treatments around the second dose of the vaccine (Supplementary Fig. S1E and S1F).

Next, we assessed whether the wane in anti-S IgG titres also correlated with waning neutralization titres (ID_50_) as might be expected. Specifically among serologic responders, we assessed the neutralization of HIV-1–based virus particles pseudotyped with SARS-CoV-2 Wuhan strain (WT) and VOC.B.1.617.2 (delta) spike at TP5 and compared these with previously reported data following completion of the two-dose vaccine schedule (refs. 1, 2; Fig. 1D and E). We observed that among all cohorts, there was a very substantial decrease in the neutralization ID_50_ for WT (HC median IC_50_, 2922.6–219.6; *P* = 0.00075; SC median IC_50_, 1110.4–302.7, *P* = 0.00028; HM median IC_50_, 379.3–117.5, *P* = 0.014; Fig. 1D), as well as substantial declines in neutralization of VOC.B.1.617.2 (delta): HC median IC_50_,1363.2to350.5, *P* = 0.0028; SC median IC_50_, 767.9 to 92.3; *P* = 0.00097; HM median IC_50_, 207.9 to 67.3, not significant (Fig. 1E).

### Waning Yet Durable Cellular Responses

**Figure 2:**
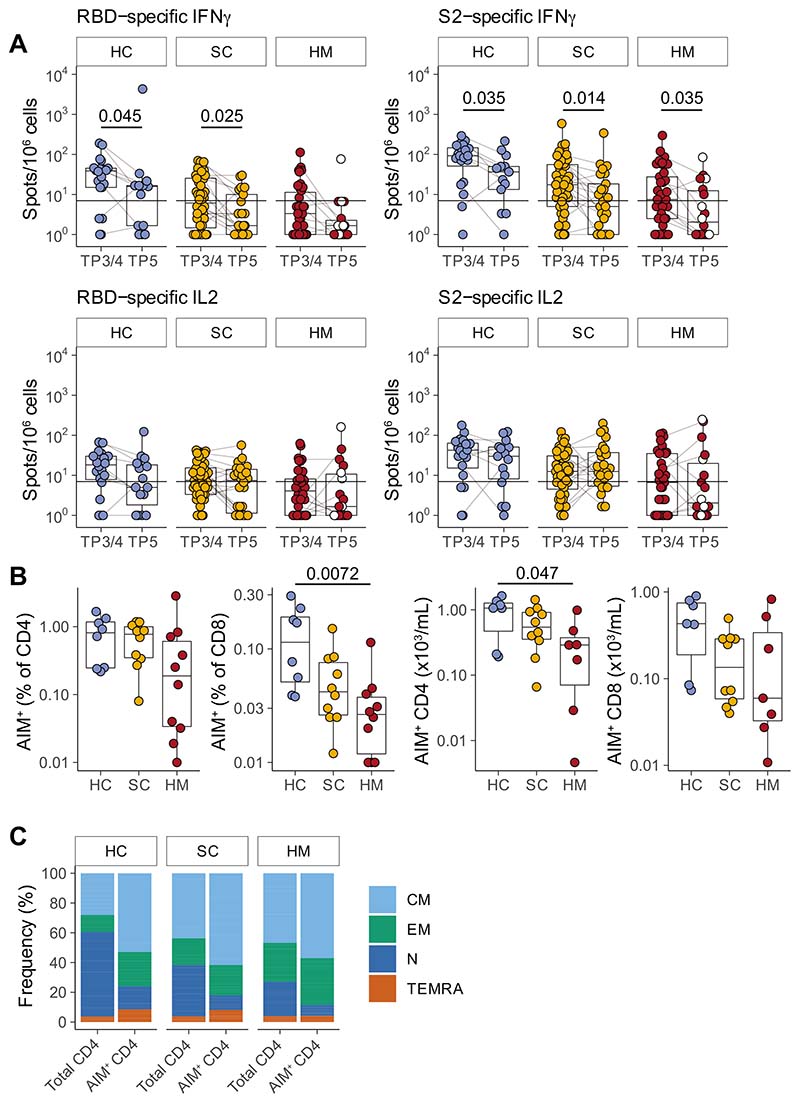
T-cell responses prior to SARS-CoV-2 vaccination dose 3. **A,** Functional T-cell responses were measured using fluorospot assays to measure IFNγ and IL2 release by T cells stimulated with one of the following peptide mixes presented on autologous antigen-presenting cells: SARS-CoV-2 spike 2 peptides (“S2”); SARS-CoV-2 RBD peptides (“RBD”); and control peptides derived from CEFT at TP3/4 and TP5. Individuals were classified as responders if they scored >7 cytokine secreting cells/10^6^ PBMC for IFNγ and/or for IL2 in response to RBD and/or S2 peptide pools. Sample comparisons tested by a partially matched Wilcoxon test and *P* values corrected by the Benjamini–Hochberg method (TP3/4: HC *n* = 20, SC *n* = 48, HM *n* = 37; TP5 HC *n* = 14, SC *n* = 26, HM *n* = 18; *n* is variable due to technical dropouts). White: MDS. **B,** Frequency (left) and number (right) of spike-specific AIM+ CD4 and CD8 T cells at TP5, as defined by AIM+ cell frequency following stimulation with spike peptide pools minus control stimulation. Sample comparisons tested by a Kruskal–Wallis test with Dunn multiple comparisons test and corrected by the Holm method (HC *n* = 8, SC *n* = 10, HM *n* = 10; *n* is variable due to cell count dropouts). **C,** Frequency of naïve and memory subsets among total and AIM+ CD4 T cells following restimulation with spike peptide pools. Boxplots represent the median, Q1 and Q3. Horizontal lines represent response thresholds. AIM: activation-induced markers; HC: healthy control; HM: hematologic malignancy; MDS: myelodysplastic syndrome; ns: nonsignificant; SC: solid cancer; TP: timepoint.

Next, we measured the functional T-cell responses to vaccination in subcohorts of individuals (HC, 14/22; SC, 29/50; and HM, 18/35) using fluorospot assays [as described previously (ref. 1; Fig. 2A)]. We observed that the rates for making SARS-CoV-2–specific IFNγ or IL2 T-cell responses to spike or to RBD at TP5 were: 79% (11/14) of HCs; 79% (23/29) of patients with SC; and 39% (7/18) of patients with HM (Fig. 2A; Supplementary Fig. S2A; Table 2). Of note, as for the antibody responses, there were significant decays in SARS-CoV-2–specific IFNγ responses at TP5 when compared with TP3/4 (2 weeks following second vaccine inoculation), the only exception being responses of patients with HM toward spike, and for those there was a clear downward trend. Interestingly there was no decline in IL2 responses, a finding that emphasizes the importance of assessing several parameters in assaying T-cell responses (Fig. 2A; Table 2).

To gain insight into whether COVID-19 vaccination induced durable antigen-specific memory T-cell responses, we performed flow cytometric analyses using an AIM assay on a subset of patients within each cohort (HC, 8; SC, 10; HM, 10) at TP5. The clinical characteristics of this group of patients are shown in Supplementary Table S3. We found that all patient PBMCs demonstrated detectable frequencies and numbers of AIM^+^ CD4^+^ cells while all but 1 patient with HM also harbored AIM^+^ CD8^+^ T cells (Fig. 2B; Supplementary Fig. S2B). In general, there was a trend for higher frequencies to be observed in HC compared with patients with cancer (Fig. 2B)at 6 months following the second dose of the vaccine, although this only reached statistical significance when the frequency of AIM^+^CD8^+^ cells and the absolute numbers of AIM^+^CD4^+^ T cells were compared across HCs and patients with HM. In sum, durable robust cellular immune responsiveness toward WT SARS-CoV-2 was retained for at least 6 months after mRNA vaccination, but clearly the capacity to elicit T-cell activation often did not translate into functional readouts, as evidenced by comparing the AIM data with the fluorospot data (14). We also characterized the differentiation status of antigen-specific CD4^+^ T cells induced by vaccination, observing that within peripheral blood, AIM^+^ CD4^+^ T cells at TP5 were enriched in CM (CCR7^+^CD45RO^+^) cells with some enrichment for EM (CCD7^−^CD45RO^+^) cells (Fig. 2C).

### Significant Serologic and Cellular Response Boosting Following Third Dose

**Figure 3:**
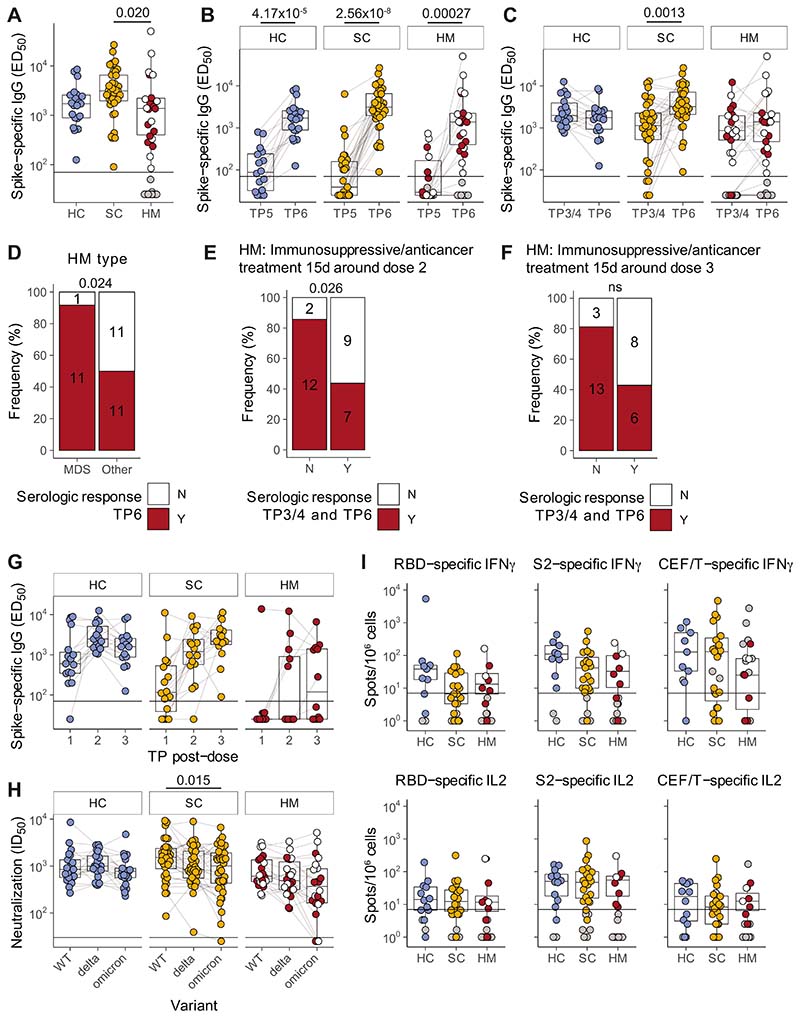
Boosted serologic responses and persistent T-cell responses following SARS-CoV-2 vaccination dose 3. **A,** Plasma spike-specific IgG titres at TP6. Numbers represent the frequency of individuals with titres above the seropositivity threshold. Sample comparisons tested by a Kruskal–Wallis test with Dunn multiple comparisons test and corrected by the Holm method. Boxplots and statistics summarize responder values only (HC *n* = 21/21, SC *n* = 37/37, HM *n* = 22/34). Gray: nonresponders; white: MDS. Spike-specific IgG titres in plasma samples at TP5 to TP6 (**B**) and TP3/4 to TP6 (**C**). Patient-matched samples are linked by lines. Matched samples at both TP were compared by a Wilcoxon signed-rank test and *P* values corrected by the Benjamini–Hochberg method. Boxplots and statistics summarize only patients who were serologic responders at both TP in (HC *n* = 15/15, SC *n* = 27/27, HM *n* = 10/18; **B**)and (HC *n* = 18/18, SC *n* = 35/35, HM *n* = 21/32; **C**). Gray: serologic nonresponders at both TP; white: MDS. **D,** Serologic response at TP6 comparing MDS and other patients with HM. Contingency analysis performed by a Fisher exact test (MDS *n* = 12, other *n* = 22); numbers on graph indicate patient counts. Serologic responses at TP6 comparing overall serologic response of patients with HM with matched TP3/4 and TP6 samples receiving immunosuppressive and/or antitumor treatment within 15 days either side of dose 2 (**E**)or dose 3 (**F**). Contingency analysis performed by a Fisher exact test in (untreated *n* = 14, treated *n* = 16; **E**)and (untreated *n* = 16, treated *n* = 14; **F**); numbers on graphs indicate patient counts. **G,** Longitudinal analysis of spike-specific IgG titres after each dose (TP2, TP3/4, and TP6) in patients with matched datasets (HC *n* = 15, SC *n* = 16, HM *n* = 12). **H,** Plasma neutralization titres against wildtype, B.1.617.2 (delta) and BA.1 (omicron) SARS-CoV-2 variants in serologic responders at TP6 (matched patient samples are linked). Sample comparisons tested by a Friedman test with Wilcoxon signed-rank post-tests and *P* values corrected by the Bonferroni method (HC *n* = 21, SC *n* = 36, HM *n* = 22). White: MDS. **I,** T-cell IFNγ and IL2 responses to SARS-CoV-2 RBD and S2 peptide pools at TP6. Only patients who were overall T-cell responders are represented by boxplots and compared by a Kruskal–Wallis test with Dunn multiple comparisons test and corrected by the Holm method (HC *n* = 12/14, SC *n* = 20/26, HM *n* = 8/19), all ns. Gray: T-cell nonresponders; white: MDS. Boxplots represent the median, Q1 and Q3. Horizontal lines represent response thresholds. ED_50_: plasma dilution at 50% binding; HC: healthy control; HM: hematologic malignancy; MDS: myelodysplastic syndrome; ns: nonsignificant; SC: solid cancer; TP: timepoint.

Next,we assessed whether waned immunity could be rescued by repeated vaccine dosing. We therefore analyzed anti-S IgG titres in patients at TP6, that is, at 3 weeks after third vaccine dose. Patient characteristics and treatments are shown (Table 1; Supplementary Table S1). Median (Q1, Q3) time (days) from third dose of vaccine to blood sampling at TP6 were comparable across cohorts: HC21 (21–23.75), SC21 (21–24), and HM21 (20–25; Table 1). Among non–virus-exposed individuals, anti-S IgG titres at 3 weeks demonstrated vigorous rescue of humoral immune responses across all three cohorts: HC: *n* = 21/21 (100%), SC: *n* = 37/37(100%), and HM *n* = 22/34 (65%; Fig. 3A and B; Table 2). Interestingly, median titres among patients with SC and HM were comparable with HCs, although a statistically significant difference was observed between SC and HM responders (median HC 1,713; SC 3,115; HM 1,379), primarily reflecting the fact that HM responses were generally lower than SC responses (Fig. 3A), as was the case following both the first and second vaccinations (1, 2).

HCs and patients with HM showed comparable median titres at TP3/4 (2 weeks following the second dose) and TP6 (following the third dose), arguing that beyond rescuing immunity, there was no additional impact of the third booster *vis-à-vis* the two-dose schedule. Interestingly, this was not the case for patients with SC, who showed a statistically significant increment at TP6 (Fig. 3C), irrespective of age or tumor type (Supplementary Fig. S3A and S3B). We previously reported that among patients with SC, most nonresponders following the second vaccine dose had received chemotherapy within 15 days of vaccine administration, especially if prescribed with concomitant high doses of steroid therapy (2). Although all patients with SC were serologic responders following the third dose, we nonetheless sought any association between the anti-IgG titre and anticytotoxic or steroid treatment within 15 days of the third dose but, of note, we did not observe any correlation with any potentially immunosuppressive treatments around the time of the third dose (Supplementary Fig. S3C and S3D).

Given that MDS accounted for approximately 35% of all patients with HM at TP6, it was possible to note that for most of these patients the third vaccine dose induced very strong boosting of their severely waned TP5 responses (Fig. 3B–D). Conversely, 50% of the patients with non-MDS HM (*n* = 11/22) continued to fail to mount a serologic response despite a third dose, highlighting the continued vulnerability of this patient population (Fig. 3B–D), and emphasizing the contribution of cancer type to vaccine responsiveness. At the same time, the cancer treatment schedule also had an influence on the HM cohort. Thus, when 19 patients with HM who responded to dose 2 and dose 3 were compared with 11 patients who responded neither to dose 2 nor to dose 3, there was a trend by which nonresponsiveness was associated with receiving anti-cancer treatment within 15 days of either dose 2 or dose 3 (Fig. 3E and F), and this trend existed even when patients with MDS were excluded from the comparison (Supplementary Fig. S3E and S3F).

Finally, several individuals across all three cohorts (HC *n* = 15, SC *n* = 16, HM *n* = 12) had blood sampled following each of three vaccinations, that is, at TP2, TP3/4, and TP6, thus providing a rare opportunity to track incremental increases in serologic responses with repeated dosing (Fig. 3G; Table 3). For HCs, all individuals had seroconverted by the time of completing their two-dose schedule. Among patients with SC, the last remaining SC nonserologic patient (receiving cytotoxic treatment around dose 2 and receiving high-dose radiotherapy within 15 days of dose 3) achieved serologic response status following the third dose. For patients with HM, the longitudinal data confirmed the influence of treatment considered above, in that of patients with non-MDS, 50% (6/12) patients failed to mount any response across three doses of vaccine (Table 3; Supplementary Table S4).

Next, among serologic responders, we assessed TP6 samples for the neutralization of HIV-1–based virus particles, pseudotyped with SARS-CoV-2 Wuhan strain (WT), VOC.B.1.617.2 (delta), and VOC BA.1 (omicron) spike. All sero-logic responders across all three cohorts could neutralize WT and delta strains (Fig. 3H). However, within the SC and HM patient cohorts, some patients exhibited conspicuously low neutralization of omicron versus WT or delta, which resulted in patients with SC considered as a whole cohort showing significantly lower neutralization of omicron versus WT. Specifically, non-neutralization values (ID_50_ of <25) were shown by four serologic responders comprising 1 patient with SC presenting with pancreatic cancer and on active cytotoxic treatment, and 3 patients with HM, of whom 2 presented with MDS and were on supportive treatments while 1 presented with Hodgkin lymphoma and was on active Adriamycin, Vinblastine Dacarbazine (AVD) chemotherapy (Fig. 3H). These exceptions notwithstanding, anti-S IgG titres of those responding to the third dose were highly correlated with neutralization among all cohorts (Supplementary Fig. S3G). Comparison of samples pre-third dose (TP5) and post-third dose (TP6) clearly demonstrated the positive effect of boosting on neutralization for all VOCs assayed across all three cohorts (Supplementary Fig. S3H).

Next, we measured functional T-cell responses to vaccination on a subset of individuals (HC *n* = 14; SC *n* = 26; HM *n* = 19). Following the third vaccine dose, SARS-CoV-2–specific IFNγ or IL2 T-cell responses to S2 or to RBD were evident for 86% (12/14) of HCs, 77% (20/26) of patients with SC and 42% (8/19) of patients with HM (Table 3; Fig. 3I). The boosting effect of the third dose on the T-cell immunogenicity (i.e., the transition from TP5 to TP6; Supplementary Fig. S3I) was not as overt as that observed in serologic and neutralization responses. In fact, for patients with HM following the third dose, very little difference was observed when comparing predose and postdose percentages of T-cell responders (39% at TP5 vs. 42% at TP6; Table 2). These observations are consistent with those made for the second vaccine administration, and they may in part reflect the prospect that T-cell responses wane less between vaccinations.

### Serologic Responses in Virus-exposed Participants

**Figure 4:**
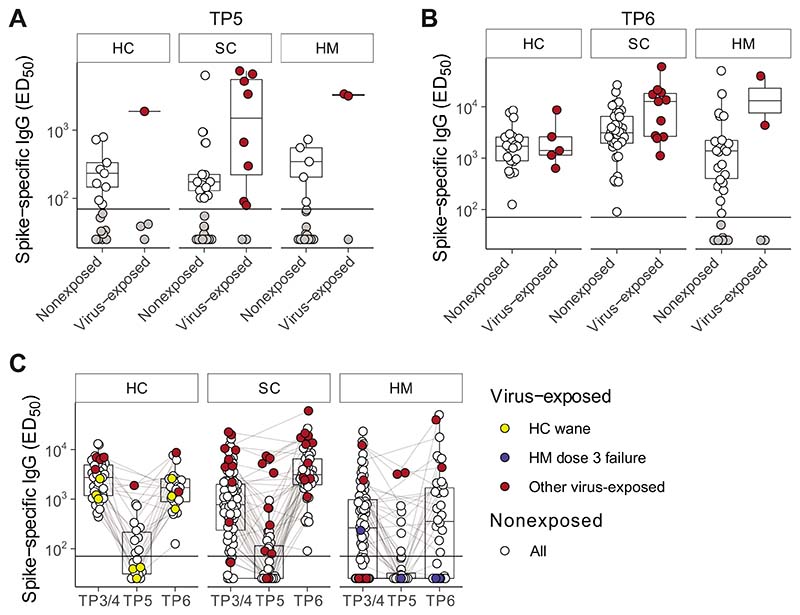
Serologic wane and boost around SARS-CoV-2 vaccine dose 3 in naturally exposed individuals. Plasma spike-specific IgG titres in unexposed and previously SARS-CoV-2 exposed patients at TP5 (**A**)and TP6(**B**). Numbers represent the frequency of individuals with titres above the seropositivity threshold. Sample comparisons tested by Wilcoxon rank-sum test and *P* values corrected by the Benjamini–Hochberg method, all ns. Boxplots and statistics summarize responder values only in (HC unexposed *n* = 9/18, exposed *n* = 1/4; SC unexposed *n* = 13/40, exposed *n* = 8/10; HM unexposed *n* = 5/32, exposed *n* = 2/3; **A**) and (HC unexposed *n* = 21/21, exposed *n* = 5/5; SC unexposed *n* = 37/37, exposed *n* = 11/11; HM unexposed *n* = 22/34, exposed *n* = 2/4; **B**). **C,** Longitudinal analysis of spike-specific IgG titres in all patients at TP3/4, TP5, and TP6 (HC unexposed *n* = 41, exposed *n* = 8; SC unexposed *n* = 86, exposed *n* = 18; HM unexposed *n* = 95, exposed *n* = 8 unique patients). Matched patient samples are linked. White: unexposed; red: naturally exposed; yellow: examples of naturally exposed HC (*n* = 3) who exhibited a wane/boost effect; blue: examples of naturally exposed HM (*n* = 2) who failed to respond to dose 3. Boxplots represent the median, Q1 and Q3. Horizontal lines represent response thresholds. ED_50_: plasma dilution at 50% binding; HC: healthy control; HM: hamatologic malignancy; MDS: myelodysplastic syndrome; ns: nonsignificant; SC: solid cancer; TP: timepoint.

As described above, 17 of the evaluable patients at TP5 and 20 at TP6 were excluded from the main comparisons because of probable SARS-CoV-2 exposure (Fig. 4A and B). Interestingly, we did not observe any statistically significant differences in the anti-S IgG titres between virus-exposed versus nonexposed serologic responders at either TP5 (Fig. 4A)or TP6 (Fig. 4B). Although there was a trend at TP5 for patients with virus-exposed SC to show spike-specific Ig titres higher than the median titres for nonexposed individuals, there were other examples of virus-exposed individuals displaying low serologic responses at TP5, and in particular three HCs with prior virus exposure demonstrated a profound wane in anti-IgG titres by 6 months following the second dose (Fig. 4C, yellow dots). Although they all mounted robust serologic responses to the third dose (Fig. 4C), there were 2 patients with HM who failed to mount any serologic response at TP6 despite their combination of three vaccine doses and evidence of virus exposure (Fig. 4C, blue dots; Supplementary Table S4). These observations clearly highlight the challenge faced by some patients with HM in mounting immune responses to SARS-CoV-2, consistent with our prior SOAP-01 study of COVID-19 in patients with cancer (11).

Finally, toxicity data were available for 145 participants (29 HC, 63 SC, 53 HM) following the third dose. In contrast to our previous observations (1, 2), we noted that the HC cohort experienced much less toxicity than patients with cancer following the third BNT162b2 vaccine dose (no toxicity: HC 93%, SC 68%, HM 75%; Supplementary Fig. S4A). Most symptomatic HC complained of fatigue as compared with patients with cancer where pain at the injection site within 7 days after injection of the third dose of the vaccine was the most commonly reported reaction (Supplementary Fig. S4B). Most of the adverse reactions were considered tolerable with an average recovery time of 48–72 hours. Follow-up of patients from the previous cohorts (1, 2, 11) has not identified any new side effects since last reporting.

## Discussion

How durable is the SARS-CoV-2 vaccine protection offered by sequential vaccinations, and what is the efficacy of boosting, particularly in patients with cancer who are generally regarded as immunocompromised, remain key clinically relevant questions. Here, we present the results of the continuation of the SOAP studies in which we have previously studied the impacts of SARS-CoV-2 infection and of successive rounds of SARS-CoV-2 vaccination respectively. Specifically, we provide data on the immune status of the SOAP cohorts at up to 9 months following the second vaccine dose (mRNA BNT162b2 or ChAdOx1 nCov-19), and the subsequent effects of a third vaccination.

A key strength and differentiating feature of our study compared with other similar studies (reviewed in ref. 8) is that results reported here offer a unique longitudinal insight into the impact of three SARS-CoV-2 vaccinations on humoral and cellular immunity in patients with SCs, patients with HMs, and persons without cancer. Following two doses of vaccine and prior to a third dose, the SARS-CoV-2 immune responses of most patients with cancer and persons without cancer had waned significantly. Specifically, 68% of patients with SC and 84% of patients with HM showed no detectable spike-specific antibodies with patients with HM demonstrating a particularly steep decline in antibody titres over time. Correlating with this was a starkly reduced capacity to effect virus neutralization. T-cell effector responses, measured as IFNγ secreting cell frequencies, also waned, although interestingly, this seemed not to be the case for IL2-secreting cells. The implication of this is that over time, the numbers of SARS-CoV-2 antigen-reactive T cells may not decline so appreciably, but their capacity to release cytokines with effector potential against virus-infected cells is diminished. Hence, a major role of vaccine boosting may be to redirect existing SARS-CoV-2–reactive T cells toward functions effective against virus-infected cells. Such insights may add to future vaccine optimization.

Our data clearly demonstrate the impactful effects of boosting on most individuals examined; for example, the frequencies of individuals demonstrating anti-S IgG titres above background jumped from 50% to 100% for HCs; from 32% to 100% for patients with SC; and reached 92% for patients with MDS. However, it only reached 50% for patients with non-MDS HM. As was true following earlier vaccinations, S-specific IgG titres correlated strongly with the neutralization of WT SARS-CoV-2 and of SARS-CoV-2 VOCs delta and omicron (BA.1). Hence, the failure of a substantial fraction of patients with HM to either seroconvert or to make strong antibody responses is an understandable cause for concern in relation to the patients’ vulnerability in open societies with high virus transmission. Thus, shaping public health policy can be informed by better understanding the vaccine responses of patients with HM, and in that regard our study makes three clear points. First, almost all patients presenting with MDS made strong responses to vaccination, suggesting that they *per se* are not in a highly vulnerable group. Second, for many patients with other types of HMs, there was little evidence that their failure to seroconvert in response to primary or secondary vaccinations would be somehow overcome by additional boosting. Hence, other means of improving their protection need to be considered. And third, the repeated failure of several patients with non-MDS HM to respond was strongly associated with receiving cancer treatment within a time frame of 1 month centred on the day of vaccination. Indeed, many such patients received treatment within 15 days before and within 15 days after the booster dose. Hence, while not questioning the importance of anticancer treatment regimens, scheduling them at greater intervals on either side of vaccination may permit better vaccine-mediated protection against SARS-CoV-2 or any other broadly circulating viruses, for example, influenza, for which vaccines are routinely available.

Several shortcomings of our study need to be acknowledged. First, the cohort of patients is relatively heterogeneous, making it difficult to generalize the overall response results within subgroups; and the overall number of subjects is limited compared with epidemiologic or phase III clinical trials. Second, it is possible that the timepoints in this study do not perfectly capture the full kinetics of the response for each individual immune component. For example, it is possible that antibody levels stabilize at timepoints beyond 9 months rather than continuing to decay at the observed rates. Moreover, we measured neither systemic nor bronchioalveolar IgA which may be important reservoirs of protection. Third, while our AIM assay is effective at capturing T-cell peak responses after vaccination, it may not be sensitive enough to detect a very low frequency of memory CD8^+^ T cells at timepoints long after vaccination. Finally, our neutralization experiments were focused on evaluating responses against HIV-1–based virus particles, pseudotyped with SARS-CoV-2 WT, VOC.B.1.617.2 (delta), and VOC BA.1 (omicron). The WT and delta variants were critical to include in this study to allow longitudinal evaluation of responses. The omicron BA.1 was a more clinically relevant variant at the time of evaluation of these assays. However, for future research, it would be important to evaluate the newer and current circulating subvariants such as BA.2.7.5, BA.4, and BA.5. Despite these shortcomings, our study contributes important data to the complex assessment of health risks and benefits of maintaining access to COVID-19 vaccine boosters amongst particular subgroups of patients with cancer.

## Supplementary Material

Supplementary Fig. S1

Supplementary Fig. S2

Supplementary Fig. S3

Supplementary Fig. S4

Supplementary Table S1

## Figures and Tables

**Table 1:** Clinical characteristics of patients evaluable for analysis at TP5 and TP6

	Healthy controls		Solid cancers		Hematologic malignancies	
	TP5	TP6	TP5	TP6	TP5	TP6
***Total numbers***	22	26	50	48	35	38
**Age**						
*Median (Q1–Q3) years*	41 (34.25–49.75)	41 (33.25–49.75)	70 (55–74)	71 (52–74)	66 (57–70)	66 (59–70)
**Sex**						
*Male*	11/22 (50%)	13/26 (50%)	18/50 (36%)	17/48 (35%)	19/35 (54%)	27/38 (71%)
*Female*	11/22 (50%)	13/26 (50%)	32/50 (64%)	31/48 (65%)	16/35 (46%)	11/38 (29%)
**Race**						
*Caucasian*	13/22 (59%)	16/26 (62%)	33/50 (66%)	34/48 (71%)	17/35 (49%)	22/38 (58%)
*BAME*	9/22 (41%)	10/26 (38%)	13/50 (26%)	9/48 (19%)	5/35 (14%)	7/38 (18%)
*Unspecified*			4/50 (8%)	5/48 (10%)	13/36 (37%)	9/38 (24%)
**Tumor types**						
*Women’s cancers (gynecological, breast)*			20/50 (40%)	17/48 (35%)		
*Urological cancers (renal, prostate, bladder)*			5/50 (10%)	5/48 (10%)		
*Skin cancers (melanoma, Merkel cell)*			5/50 (10%)	5/48 (10%)		
*Thoracic malignancies (lung, mesothelioma)*			7/50 (14%)	4/48 (8%)		
*GI cancers*			11/50 (22%)	13/48 (27%)		
*Head and neck cancers*			2/50 (4%)	2/48 (4%)		
*Brain cancers*			–	2/48 (4%)		
Mature B-cell neoplasms					17/35 (49%)	20/38 (53%)
*Chronic lymphocytic leukemia*					*2*	*3*
*Plasma cell Myeloma*					*8*	*10*
*Diffuse large B-cell lymphoma*					*0*	*1*
*Follicular lymphoma*					*2*	*2*
*Mantle cell lymphoma*					*1*	*1*
*MALT lymphoma*					*2*	*1*
*Hodgkin lymphoma*					*1*	*1*
*Post-transplant lymphoproliferative disorder*					*1*	*1*
Mature T-cell neoplasms					2/35 (6%)	2/38 (5%)
*Anaplastic large cell lymphoma*					*2*	*1*
*Angioimmunoblastic T-cell lymphoma*					*0*	*1*
Myeloid and acute leukemia neoplasm					15/35 (43%)	15/38 (39%)
*Myelodysplastic syndrome (MDS)*					*14* ^a^	*13* ^b^
*Acute myeloid leukemia*						
*Chronic myelomonocytic leukemia*					*0*	*1*
*T-cell acute lymphoblastic leukemia*						
*Myelofibrosis*					*1*	*1*
Others					1/35 (3%)	1/38 (3%)
*Amyloid light-chain (AL) amyloidosis*					*1*	*1*
**TNM staging**^a^ **(solids only)**						
*1*			5/50 (10%)	4/48 (8%)		
*11*			6/50 (12%)	7/48 (15%)		
*111*			11/50 (22%)	11/48 (23%)		
*1V*			27/50 (54%)	23/48 (48%)		
*Missing data*			1/50 (2%)	3/48 (6%)		
**Time from cancer diagnosis to study recruitment**						
*<3 months*			9/50 (18%)	7/48 (15%)	4/35 (11%)	3/38 (8%)
*3–12 months*			9/50 (18%)	12/48 (25%)	5/35 (14%)	7/38 (18%)
*12–24 months*			16/50 (32%)	13/48 (27%)	2/35 (6%)	4/38 (11%)
*>24 months*			14/50 (28%)	14/48 (29%)	19/35 (54%)	21/38 (55%)
*Missing info*			2/50 (4%)	2/48 (4%)	5/35 (14%)	3/38 (8%)
**Median time from dose 2 to blood sampling at TP5**						
*Median (Q1–Q3) days*	215.5 (200–259)		187 (180–213)		188 (180.5–196)	
**Median time from dose 3 to blood sampling at TP6**						
*Median (Q1–Q3) days*	21 (21–23.75)		21 (21–24)		21 (20–25)	

NOTE: TP5 = blood sampling prior to the third dose; TP6 = 3 weeks after the third vaccine dose of the BNTl62b2 vaccine.

a 12/14 patients with TP5 sample available had received ChAdOx1 nCov-19 as the first and/or second dose.

b 9/13 patients with TP6 sample available had received ChAdOx1 nCov-19 as the first and/or second dose.

**Table 2:** Waning immune efficacy following two doses of COVID-19 vaccines at 3 weeks after dose (TP3/4), prior to receiving the third dose of BNT162b2 vaccine (>6 months after dose 2; TP5), and 3 weeks after third dose of BNT162b2 vaccine (TP6)

	Healthy controls			Solid cancers			Hematologic malignancies		
	TP3/4	TP5	TP6	TP3/4	TP5	TP6	TP3/4	TP5	TP6
Anti-SARS-CoV-2 IgG response	100%	50%	100%	87%	32%	100%	61%	16%	65%
	(91–100)	(29–71)	(85–100)	(78–92)	(20–48)	(91–100)	(51–71)	(7–32)	(48–79)
	(38/38)	(9/18)	(21/21)	(72/83)	(13/40)	(37/37)	(57/93)	(5/32)	(22/34)
T-cell vaccine response	90%	79%	86%	92%	79%	77%	70%	39%	42%
	(70–97)	(52–92)	(60–96)	(80–97)	(62–90)	(58–89)	(54–83)	(20–61)	(23–64)
	18/20	(11/14)	12/14	44/48	(23/29)	20/26	26/37	(7/18)	8/19
Anti-SARS-CoV-2 IgG response and T-cell vaccine response	90%	50%	86%	79%	35%	77%	41%	11%	32%
	(70–97)	(27–73)	(60–96)	(66–88)	(19–54)	(58–89)	(26–57)	(3–33)	(15–54)
	18/20	7/14	12/14	38/48	9/26	20/26	15/37	2/18	6/19

NOTE: Percentages in parentheses represent 95% confidence intervals calculated by the Wilson method.

**Table 3:** Longitudinal serologic response data patients with blood sampling captured following each vaccine dose

	Healthy controls	Solid cancers	Hematologic malignancies
Serologic response gained following dose 1	14/15; 93% (70–99)	11/16; 69% (44–86)	1/12; 8% (1–35)
Serologic response gained following dose 2	1/15; 7% (25–70)	4/16; 25% (10–49)	4/12; 33% (14–61)
Serologic response gained following dose 3	–	1/16; 6% (1–28)	1/12; 8% (1–35)
Failed serologic response following three doses	–	–	6/12; 50% (25–75)

## Significance

Global health policy reliant on SARS-CoV-2 vaccine effectiveness is underpinned by our understanding of the durability of protection offered by sequential vaccinations and the efficacy of boosting, especially in immunocompromised patient populations who might constitute virus reservoirs. Here, we have: (i) clarified in patients with cancer the degree of waning of antibodies, serum neutralization titres against parental virus and variants of concern, and T-cell responses; (ii) evaluated the immune response among patients with cancer to a third dose of COVID-19 vaccine; and (iii) provided safety data following the third dose of the BNT162b2 COVID-19 vaccine in patients with cancer.
