# Supplementary figures and images for: Boosting of Waned Humoral and Cellular Responses to SARS-CoV-2 Variants of Concern Among Patients with Cancer
Source: Cancer Res Commun. 2022 Nov 17;2(11):1449–61. doi: 10.1158/2767-9764.CRC-22-0298 (PMC7614214; doi:10.1158/2767-9764.CRC-22-0298)

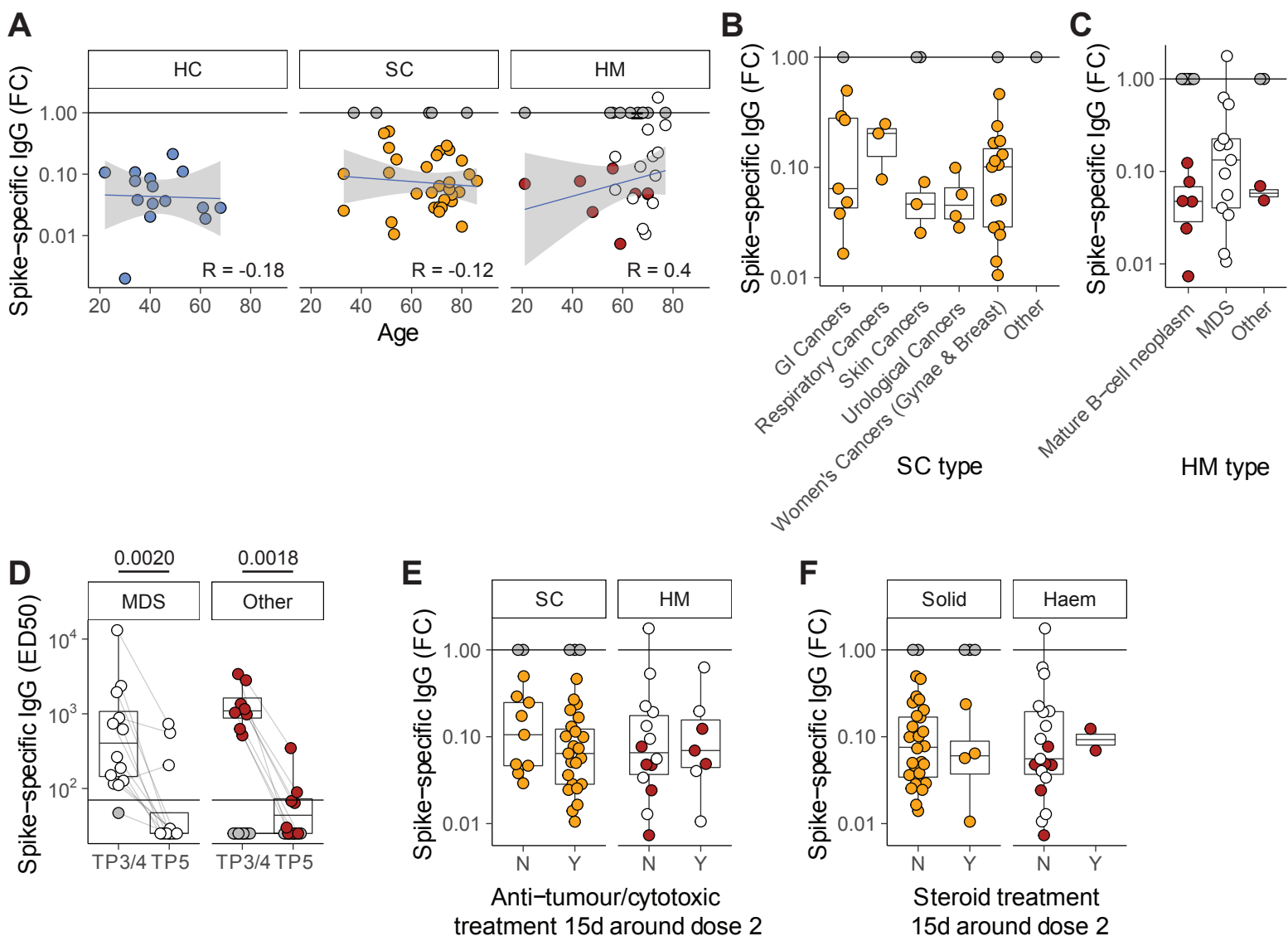

Supplementary Figure 1

Supplement: Figure S1 — S1. Waning serological responses to SARS-CoV-2 vaccination. [file crc-22-0298-s01.pdf]

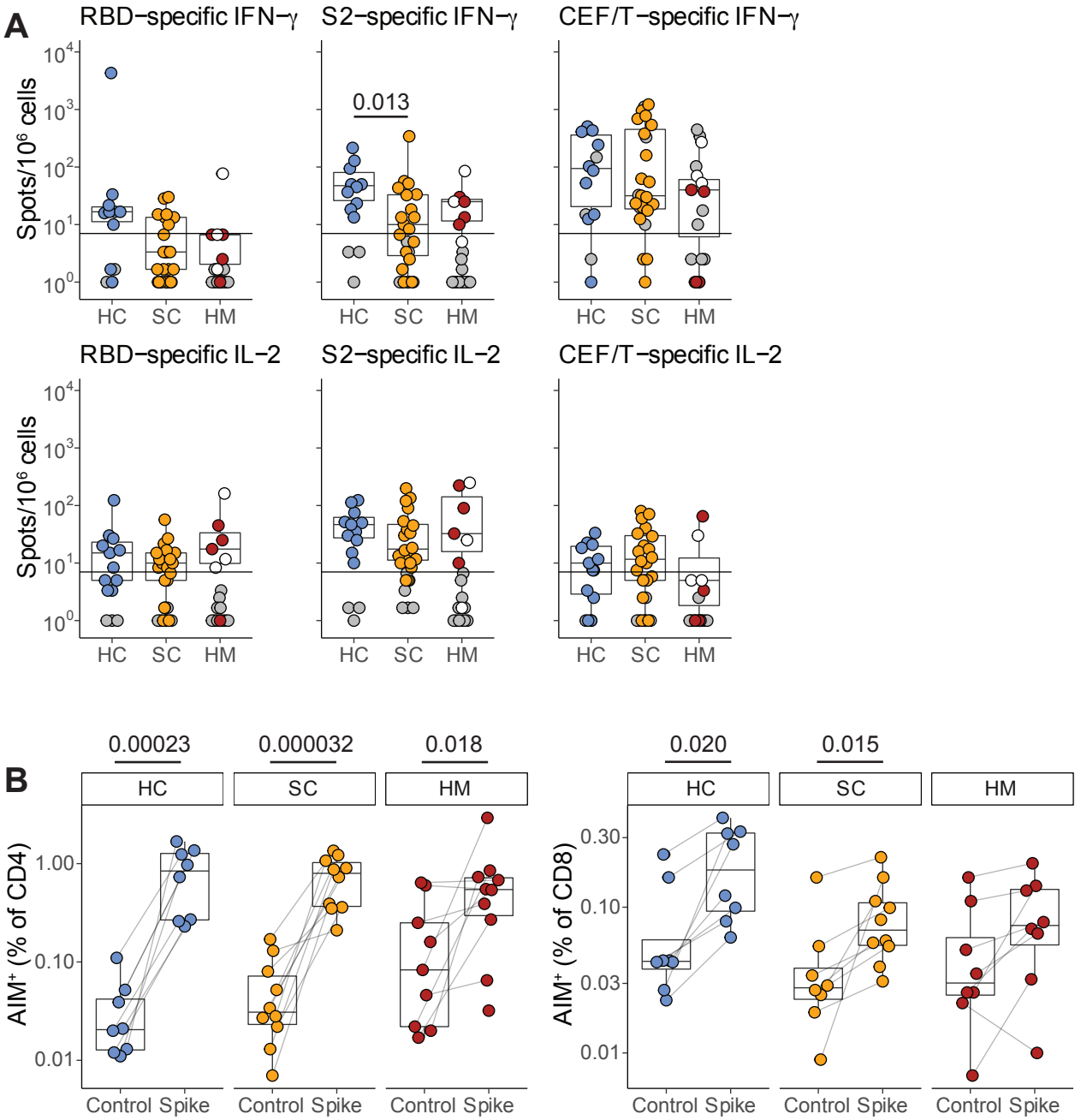

Supplementary Figure 2

Supplement: Figure S2 — S2. T cell responses prior to SARS-CoV-2 vaccination dose 3. [file crc-22-0298-s02.pdf]

**A**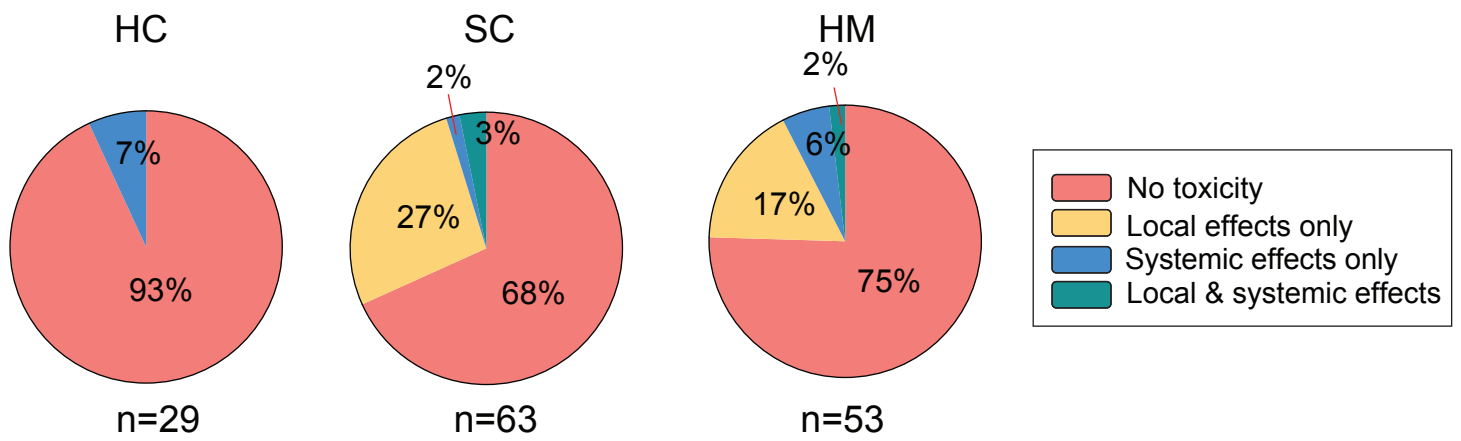**B**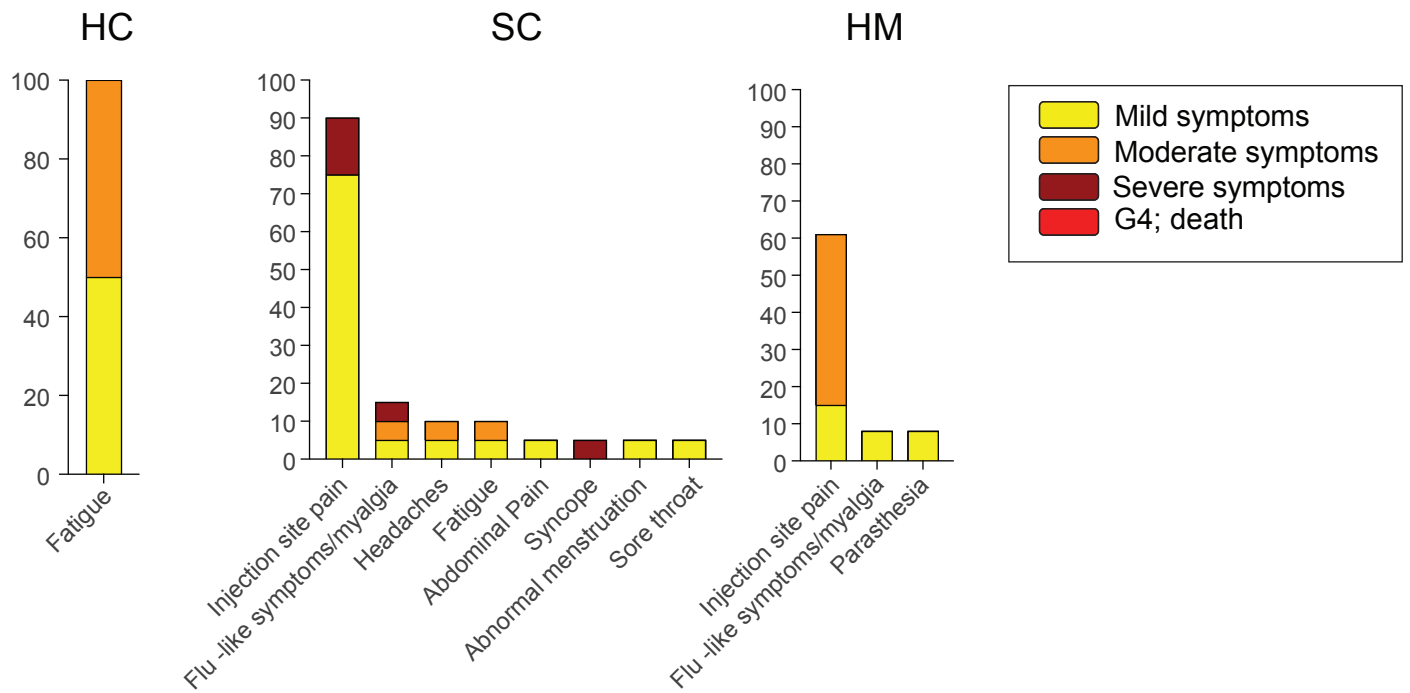

Supplement: Figure S4 — S4. Local and systemic effects reported within 30 days after 3rd dose of COVID-19 vaccine in patients with solid and haematological cancers and healthy controls. [file crc-22-0298-s04.pdf]
