# Supplementary material for: Boosting of Waned Humoral and Cellular Responses to SARS-CoV-2 Variants of Concern Among Patients with Cancer
Source: Cancer Res Commun. 2022 Nov 17;2(11):1449–61. doi: 10.1158/2767-9764.CRC-22-0298 (PMC7614214; doi:10.1158/2767-9764.CRC-22-0298)
Supplement: Supplementary Data SD1 — Supplementary Table & Figure Legends [file crc-22-0298-s05.docx]

**Supplementary Tables**

**Supplementary Table 1: Anti-cancer treatments prior to or after administration of the 3^rd^ dose vaccination for patients evaluable at TP6.** TP6= 3-weeks after the third vaccine dose of the BNT162b2 vaccine

| Solid Cancers (n=48) | | | |
| --- | --- | --- | --- |
|  | 0-15 days | 16-29 days | >30-days |
| Prior to delayed vaccine boost | **20/48 (42%)** | **2/48 (4%)** | **26/48 (54%)** |
| *Treatment naïve/no treatment* | 1/20 (5%) | 0/2 (0%) | 7/26 (27%) |
| *Targeted therapies alone* | 5/20 (25%) | 0/2 (0%) | 1/26 (4%) |
| *Chemotherapy +/- targeted therapies* | 6/20 (30%) | 1/2 (50%) | 8/26 (31%) |
| *Immune Checkpoint inhibition (ICI)* | 1/20 (5%) | 1/2 (50%) | 1/26 (4%) |
| *Chemotherapy/targeted + ICI* | 2/20 (10%) | 0/2 (0%) | 0/26 (0%) |
| *Radiotherapy* | 0/20 (0%) | 0/2 (0%) | 6/26 (23%) |
| *Other (endocrine/surgery)* | 5/20 (25%) | 0/2 (0%) | 3/26 (12%) |
| Post delayed vaccine boost | **23/48 (48%)** | **3/48 (6%)** | **22/48 (46%)** |
| *Treatment naïve/no treatment* | 1/23 (4%) | 0/3 (0%) | 18/22 (82%) |
| *Targeted therapies* | 5/23 (22%) | 0/3 (0%) | 0/22 (0%) |
| *Chemotherapy +/- targeted therapies* | 6/23 (26%) | 2/3 (67%) | 2/22 (9%) |
| *Immune Checkpoint inhibition (ICI)* | 2/23 (9%) | 0/3 (0%) | 1/22 (5%) |
| *Chemotherapy/targeted + ICI* | 1/23 (4%) | 1/3 (33%) | 0/22 (0%) |
| *Radiotherapy* | 0/23 (0%) | 0/3 (0%) | 0/22 (0%) |
| *Other (endocrine/surgery)* | 8/23 (35%) | 0/3 (0%) | 1/22 (5%) |
| Haematological Cancers (n=38) | | | |
|  | 0-15 days | 16-29 days | >30-days |
| Prior to delayed vaccine boost | **15/38 (39%)** | **1/38 (3%)** | **22/38 (58%)** |
| *Treatment naïve/no anti-cancer treatment* | 0/15 (0%) | 0/1 (0%) | 15/22 (68%) |
| *Chemotherapy +/- Targeted therapies** | 0/15 (0%) | 0/1 (0%) | 2/22 (9%) |
| *Chemo/targeted therapies + immunotherapy** | 1/15 (7%) | 0/1 (0%) | 0/22 (0%) |
| *Single agent monoclonal antibody* | 2/15 (13%) | 0/1 (0%) | 2/22 (9%) |
| *Targeted therapies** | 12/15 (80%) | 1/1 (100%) | 2/22 (9%) |
| *Radiotherapy* | 0/15 (0%) | 0/1 (0%) | 1/22 (5%) |
| Post delayed vaccine boost | **14/38 (37%)** | **3/38 (8%)** | **21/38 (55%)** |
| *Treatment naïve/no anti-cancer treatment* | 0/14 (0%) | 0/3 (0%) | 19/21 (90%) |
| *Chemotherapy +/- Targeted therapies** | 0/14 (0%) | 0/3 (0%) | 0/21(0%) |
| *Chemo/targeted therapies + immunotherapy** | 1/14 (7%) | 1/3 (33%) | 0/21 (0%) |
| *Single agent monoclonal antibody* | 2/14 (14%) | 1/3 (33%) | 0/21 (0%) |
| *Targeted therapies** | 11/14 (79%) | 1/3 (33%) | 2/21 (10%) |
| *Radiotherapy* | 0/14 (0%) | 0/3 (0%) | 0/21(0%) |

**Chemotherapy regimens include mini-CHOP, hydroxycarbamide, mercaptopurine, methotrexate; targeted therapies include BTKi, Bcl2i, bortezomib; monoclonal antibodies include: Anti-CD30, Anti-CD20; Chemo/targeted therapies + immunotherapies include combination therapies with anti-CD20, Anti-CD30, Anti-CD38 therapies.*

**Supplementary Table 2: Change in spike serological response following 2 doses of COVID-19 vaccine to TP5 in patients with matched evaluable samples**

|  | **Healthy Controls** | **Solid Cancers** | **Haematological Cancers** |
| --- | --- | --- | --- |
| Loss of serological response to below cut-off | 8/15; 53% (30-75) | 19/37; 51% (36-67) | 15/31; 48% (32-65) |
| Unchanged serological response | 7/15; 47% (25-70) | 18/37; 49% (33-64) | 16/31; 52% (35-68) |
| *Remain serological responder* | 7/7; 100% (65-100) | 12/18; 67% (44-84) | 5/16; 31% (14-56) |
| *Remain non-serological responder* | 0/7; 0% (0-35) | 6/18; 33% (16-56) | 11/16; 69% (44-86) |

**Supplementary Table 3: Clinical characteristics of T cell stimulation assay cohort**

|  | Healthy Controls | Solid Cancers | Haematological Cancers |
| --- | --- | --- | --- |
| *Total numbers* | 8 | 10 | 10 |
| Age | | | |
| *Median (Q1-Q3) years* | 40 (34-50) | 68.5 (56-75) | 61 (56.75-65.75) |
| Sex | | | |
| *Male* | 6/8 (75%) | 4/10 (40%) | 6/10 (60%) |
| *Female* | 2/8 (25%) | 6/10 (60%) | 4/10 (40%) |
| Race | | | |
| *Caucasian* | 4/8 (50%) | 7/10 (70%) | 6/10 (60%) |
| *BAME* | 4/8 (50%) | 3/10 (30%) | 3/10 (30%) |
| *Unspecified* |  |  | 1/10 (10%) |

**Supplementary Table 4: Breakdown of non-serological responders despite 3 doses of COVID19 vaccines**

| **Cancer subgroup** | | **Age** | **Treatment within 15 days of priming inoculum** | **Treatment within 15 days of 2^nd^ dose** | **Treatment within 15 days of 3^rd^ dose** |
| --- | --- | --- | --- | --- | --- |
| **Non-virus exposed individuals** | | | | | |
| Mature B -cell neoplasms | Plasma cell Myeloma | 63 | Lenalidomide | Lenalidomide | Lenalidomide |
|  | Plasma cell Myeloma | 75 | None | None | Lenalidomide |
|  | Mantle cell lymphoma | 68 | BTK inhibitor | BTK inhibitor | BTK inhibitor |
|  | DLBCL | 68 | R-CHOP | R-CHOP | None |
|  | CLL/SLL | 56 | BCL2 inhibitor | BCL2 inhibitor | Anti-CD20 mAb (Obinutuzumab) + BCL2 inhibitor |
| Myeloid and acute leukaemia neoplasm | Myelofibrosis | 66 | JAK inhibitor + antimetabolite | JAK inhibitor + antimetabolite | JAK inhibitor |
| **Virus exposed individuals** | | | | | |
| Mature B -cell neoplasms | Plasma cell Myeloma | 66 | None | VTD (Bortezomib, Thalidomide, Dexamethasone), Zometa | None |
| Mature T cell neoplasms | Angioimmunoblastic T-cell lymphoma | 81 | Mini-CHOP | None | Anti-CD20 mAb (Rituximab) + Lenalidomide |

**Supplementary Figure Legends**

**Supplementary Figure 1: Waning serological responses to SARS-CoV-2 vaccination. a)** Fold change in plasma Spike-specific IgG titres from TP3/4 to TP5 by age. Associations tested by a Spearman correlation; lines depict linear regression with 95% confidence intervals shaded. Only individuals with Spike titres >25 at either TP are tested (HC n=15/15, SC=31/36, HM=21/31). Grey: individuals with Spike titres<25 at both TP; white: MDS. **b-c)** Fold change in Spike-specific IgG titres from TP3/4 to TP5 by **b)** SC subtypes and **c)** HM subtypes. Only individuals with Spike titres >25 at either TP are represented by boxplots and tested by a Kruskal-Wallis test with Dunn’s multiple comparisons test and corrected by the Holm method in b) (GI cancers n=7/8, respiratory cancers n=3/3, skin cancers n=3/5, urological cancers n=4/4, women’s cancers n=15/16, other n=0/1) and c) (mature B cell neoplasm n=6/14, MDS n=13/13, other n=2/4). Grey: individuals with Spike titres<25 at both TP; white: MDS. **d)** Spike-specific IgG titres in MDS and other HM patients with matched TP3/4 and TP5 samples (linked by lines). Sample comparisons tested by Wilcoxon-signed rank test and p-values corrected by the Benjamini-Hochberg method. Only patients who were serological responders at either TP are represented in boxplots and tested (MDS n=12/13, other n=8/18). Grey: non responders at both TP; white: MDS. **e-f)** Fold change in Spike-specific IgG titres from TP3/4 to TP5 by **e)** anti-tumour/cytotoxic or **f)** steroid treatments administered within 15 days either side of dose 2. Only individuals with Spike titres >25 at either TP are represented by boxplots and tested by a Wilcoxon rank sum test and p-values corrected by the Benjamini-Hochberg method in e) (SC untreated n=9/11, treated n=23/26; HM untreated n=14/15, treated n=7/16) and f) (SC untreated n=28/30, treated n=4/7; HM untreated n=19/27, treated n=2/4); all ns. Grey: individuals with Spike titres<25 at both TP; white: MDS. Boxplots represent the median, Q1 and Q3. Horizontal lines represent a-c, e-f) fold change of 1 or d) response thresholds. HC: healthy control; SC: solid cancer; HM: haematological malignancy; TP: time point; ns: non-significant; ED50: plasma dilution at 50% binding; MDS: myelodysplastic syndrome.

**Supplementary Figure 2: T cell responses prior to SARS-CoV-2 vaccination dose 3. a)** T cell IFN-γ and IL-2 responses to SARS-CoV-2 RBD and S2 peptide pools at TP5. Only patients who were overall T cell responders are represented by boxplots and compared by a Kruskal-Wallis test with Dunn’s multiple comparisons test and corrected by the Holm method (HC n=11/14, SC n=20/26, HM n=7/18). Grey: T cell non-responders; white: MDS. **b)** Frequency of AIM+ CD4 (left) and CD8 (right) T cells from TP5 following restimulation with Spike peptide pools or control (individuals are linked). Sample comparisons tested by Wilcoxon-signed rank test and p-values corrected by the Benjamini-Hochberg method (HC n=8, SC n=10, HM n=10). Boxplots represent the median, Q1 and Q3. Horizontal lines represent response thresholds. HC: healthy control; SC: solid cancer; HM: haematological malignancy; TP: time point; ns: non-significant; AIM: activation-induced markers; MDS: myelodysplastic syndrome.

**Supplementary Figure 3: Boosted serological responses and persistent T cell responses following SARS-CoV-2 vaccination dose 3. a)** Association of plasma Spike-specific IgG titres with age at TP6. Correlations tested by a Spearman correlation; lines depict linear regression with 95% confidence intervals shaded (HC n=21, SC n=36, HM n=34). White: MDS. **b)** Spike-specific IgG titres at TP6 comparing SC subtypes (GI cancers n=11, respiratory cancers n=2, skin cancers n=4, urological cancers n=4, women’s cancers n=13, other n=3). **c-d)** Spike-specific IgG titres at TP6 in SC patients receiving treatment with **c)** anti-tumour/cytotoxic agents or **d)** steroids within 15 days either side of dose 3. Sample comparisons tested by a Wilcoxon rank sum test in c) (untreated n=15, treated n=22) and d) (untreated n=28, treated n=9), all ns.  **e-f)** Serological responses at TP6 comparing overall serological response of non-MDS HM patients with matched TP3/4 and TP6 samples receiving immunosuppressive/anti-tumour treatment within 15 days either side of **e)** dose 2 or **f)** dose 3. Contingency analysis performed by a Fisher’s exact test in e) (untreated n=6, treated n=12) and f) (untreated n=8, treated n=10); numbers on graphs indicate patient counts. **g)** Correlation between Spike-specific IgG titres and neutralisation titres against wildtype, B.1.617.2 (delta) and B.A.1 (omicron) SARS-CoV-2 variants at TP6 in serological responders. Correlations tested by a Spearman correlation; lines depict linear regression with 95% confidence intervals shaded (HC n=21, SC n=35, HM n=22). White: MDS. **h)** Plasma neutralisation titres against wildtype, B.1.617.2 (delta) and BA.1 (omicron) SARS-CoV-2 variants at TP5 and TP6. Sample comparisons tested by a partially matched Wilcoxon test and p-values corrected by the Benjamini-Hochberg method (TP5: HC n=8, SC n=9, HM n=9; TP6 HC n=21, SC n=36, HM n=28). White: MDS. **i)** T cell IFN-γ and IL-2 responses to SARS-CoV-2 RBD and S2 peptide pools or CEF/T peptide pools at TP5 and TP6. Samples compared by a Wilcoxon-signed rank test and p-values corrected by the Benjamini-Hochberg method (HC n=12, SC n=26, HM n=16), all ns. White: MDS. Boxplots represent the median, Q1 and Q3. Horizontal lines represent response thresholds. HC: healthy control; SC: solid cancer; HM: haematological malignancy; TP: time point; ns: non-significant; ED50: plasma dilution at 50% binding; ID50: inhibitory dilution at which 50% of virus particles are neutralised; MDS: myelodysplastic syndrome.

**Supplementary Figure 4: Local and systemic effects reported within 30 days after 3^rd^ dose of COVID-19 vaccine in patients with solid and haematological cancers and healthy controls.** Data on local and systemic reactions were collected via telephone consultations with participants for 30 days after vaccination. (**A**) Proportion of participants reporting no toxicity or toxicity (local effects only vs systemic effect only vs both local and systemic effects) following third dose of COVID19 vaccine. (**B**) Breakdown of specific local and systemic side-effects following the third dose. Symptoms were assessed according to the following scale: grade 1 (mild; does not interfere with activity), grade 2 (moderate; interferes with activity), grade 3 (severe; prevents daily activity), and grade 4 (potentially life-threatening; emergency department visit or admission to hospital).
