# Supplementary material for: Boosting of Waned Humoral and Cellular Responses to SARS-CoV-2 Variants of Concern Among Patients with Cancer
Source: Cancer Res Commun. 2022 Nov 17;2(11):1449–61. doi: 10.1158/2767-9764.CRC-22-0298 (PMC7614214; doi:10.1158/2767-9764.CRC-22-0298)
Supplement: Figure S3 — S3. Boosted serological responses and persistent T cell responses following SARS-CoV-2 vaccination dose [file crc-22-0298-s03.pdf]

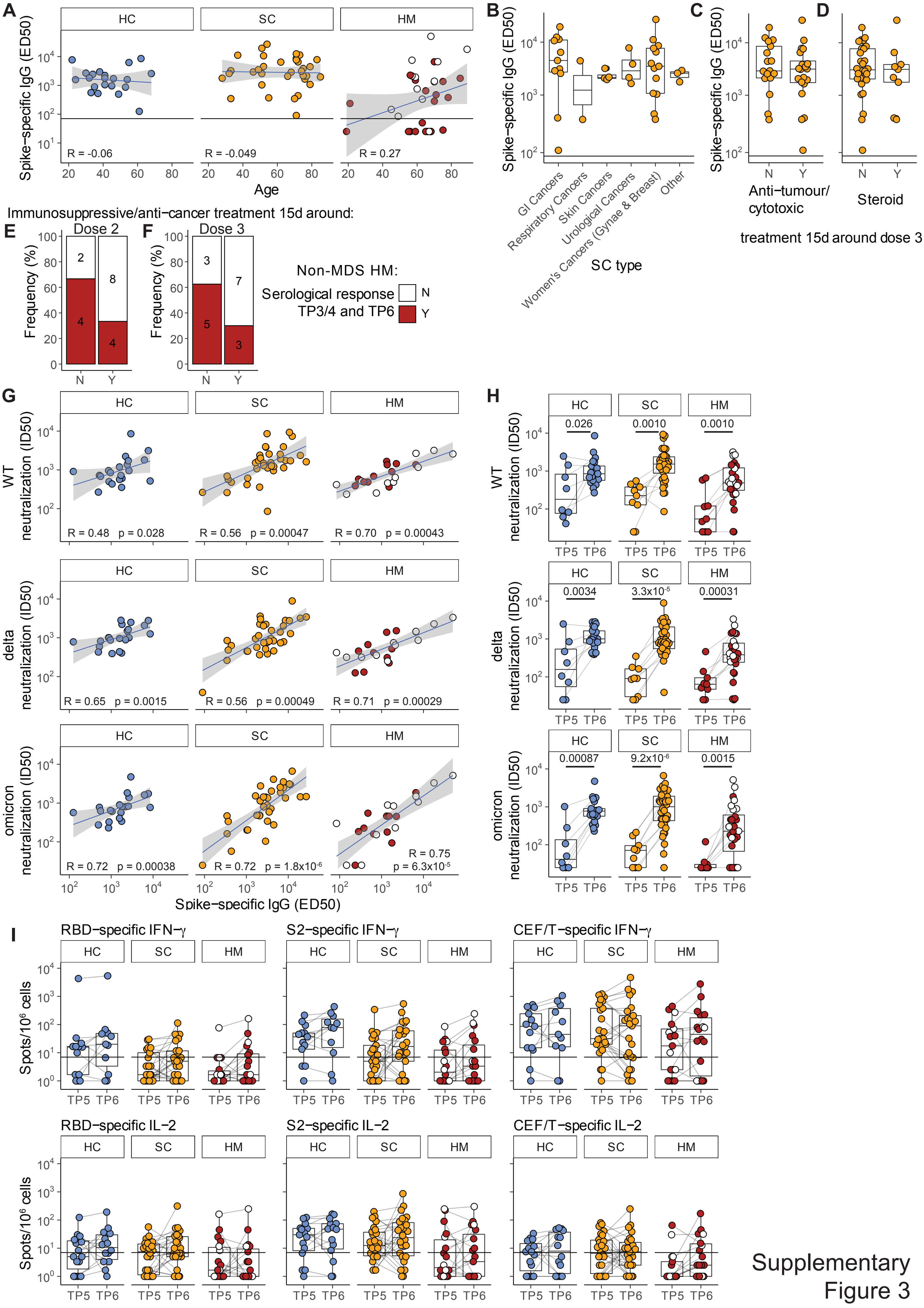

Supplementary Figure 3
